# The Phenylpropanoid Pathway and Lignin in Defense against *Ganoderma boninense* Colonized Root Tissues in Oil Palm (*Elaeis guineensis* Jacq.)

**DOI:** 10.3389/fpls.2017.01395

**Published:** 2017-08-15

**Authors:** Nisha T. Govender, Maziah Mahmood, Idris A. Seman, Mui-Yun Wong

**Affiliations:** ^1^Institute of Plantation Studies (IKP), Universiti Putra Malaysia Serdang, Malaysia; ^2^School of Environmental and Natural Resource Sciences, Faculty of Science and Technology, Universiti Kebangsaan Malaysia Bangi, Malaysia; ^3^Department of Biochemistry, Faculty of Biotechnology and Biomolecular Sciences, Universiti Putra Malaysia Serdang, Malaysia; ^4^Ganoderma and Disease Research of Oil Palm (GANODROP) Unit, Malaysian Palm Oil Board Bandar Baru Bangi, Malaysia; ^5^Department of Plant Protection, Faculty of Agriculture, Universiti Putra Malaysia Serdang, Malaysia

**Keywords:** basal stem rot, cinnamyl alcohol dehydrogenase (CAD), guaiacyl, lignin, peroxidase (POD), phenylalanine ammonia lyase (PAL), syringyl, oil palm

## Abstract

Basal stem rot, caused by the basidiomycete fungus, *Ganoderma boninense*, is an economically devastating disease in Malaysia. Our study investigated the changes in lignin content and composition along with activity and expression of the phenylpropanoid pathway enzymes and genes in oil palm root tissues during *G. boninense* infection. We sampled control (non-inoculated) and infected (inoculated) seedlings at seven time points [1, 2, 3, 4, 8, and 12 weeks post-inoculation (wpi)] in a randomized design. The expression profiles of phenylalanine ammonia lyase (PAL), cinnamyl alcohol dehydrogenase (CAD), and peroxidase (POD) genes were monitored at 1, 2, and 3 wpi using real-time quantitative polymerase chain reaction. Seedlings at 4, 8, and 12 wpi were screened for lignin content, lignin composition, enzyme activities (PAL, CAD, and POD), growth (weight and height), and disease severity (DS). Gene expression analysis demonstrated up-regulation of PAL, CAD, and POD genes in the infected seedlings, relative to the control seedlings at 1, 2, and 3 wpi. At 2 and 3 wpi, CAD showed highest transcript levels compared to PAL and POD. DS increased progressively throughout sampling, with 5, 34, and 69% at 4, 8, and 12 wpi, respectively. Fresh weight and height of the infected seedlings were significantly lower compared to the control seedlings at 8 and 12 wpi. Lignin content of the infected seedlings at 4 wpi was significantly higher than the control seedlings, remained elicited with no change at 8 wpi, and then collapsed with a significant reduction at 12 wpi. The nitrobenzene oxidation products of oil palm root lignin yielded both syringyl and guaiacyl monomers. Accumulation of lignin in the infected seedlings was in parallel to increased syringyl monomers, at 4 and 8 wpi. The activities of PAL and CAD enzymes in the infected seedlings at DS = 5–34% were significantly higher than the control seedlings and thereafter collapsed at DS = 69%.

## Introduction

Oil palm (*Elaeis guineensis*), the world’s most efficient oil-bearing tree (Gardner et al., 2010; Parveez et al., 2015) is an important economic crop in the tropics. The palm oil accounts for approximately 45% of the global edible oil production (Ooi et al., 2016) and is primarily consumed in the food industries. In addition, palm oil-based formulations are extensively incorporated in cosmetics, personal-care and household products. The oil is also an effective bioenergy feedstock (Choo et al., 2012). Methyl esters from palm oil follow the international specifications for biodiesel EN 14214214 and ASTMD 6751 (Choo and Cheah, 2000). Oil palm tree is indigenous to West Africa (Corley and Tinker, 2007) and is cultivated in 43 tropical countries throughout the world (FAO, 2016). The perennial crop grows in hot and humid conditions and the average yield ranges from 3 to 4 tons of oil per hectare per year (Wahid et al., 2005; Sumathi et al., 2008; Basiron, 2016). Nevertheless, the full economic yield potential of oil palm is estimated to range from 8 to 12 tons of oil per hectare per year under efficient cultivation practices which consider fertilization, irrigation, good planting material, favorable climate and successful disease management practices (Singh, 2014). A major challenge to agricultural research communities is to maximize production on the currently cultivated lands using cost-effective measures (Ravigadevi et al., 2009; Murphy, 2014).

Yield improvement on existing plantations confers substantial economic gain and reduces the need for further land expansion. In South East Asia, basal stem rot (BSR), caused by the pathogenic white rot fungi, *Ganoderma boninense* severely affects the productivity of oil palm. The soil-borne fungus penetrates into the root cortex with needle-like micro-hyphae (Govender and Wong, 2017), establishes within the host’s niche (biotroph stage) and finally kills (necrotroph stage) the host (Rees et al., 2009; Sundram et al., 2014). The fungus uses lignin modifying enzymes for a successful host penetration and subsequent wood degradation (Lundell et al., 2010). Although *G. boninense* pathogenesis in oil palm is well characterized, the corresponding host response in respect to lignin and lignification is less known. By inferring the role of lignin in oil palm defense response against *Ganoderma* infection, the conventional BSR management and oil palm breeding strategies would likely be improved.

Lignin is an aromatic heteropolymer synthesized through the complex phenylpropanoid metabolism. The phenylpropanoid pathway begins with the deamination of L-phenylalanine into cinnamic acid by the phenylalanine ammonia lyase (PAL) enzyme. The putative cinnamic acid undergoes a series of reduction processes catalyzed by the cinnamyl alcohol dehydrogenase (CAD) and cinnamoyl-CoA reductase (CCR) enzymes to produce hydroxycinnamyl alcohols, cumulatively known as monolignols [p-hydroxyphenyl (H), guaiacyl (G) and syringyl (S)]. Finally, polymerization of monolignols catalyzed by peroxidase (POD) and/or laccase (LAC) enzymes yields lignin (Higuchi, 1990; Boudet, 2000; Donaldson, 2001; Boerjan et al., 2003; Vanholme et al., 2008; Ralph, 2010; Tu et al., 2010; Srivastava et al., 2015).

Oil palm lignin has been extensively studied in the lignocellulosic waste and wood pulp industries, which include empty fruit bunch (Ibrahim et al., 2011; Medina et al., 2015; Nakagawa-Izumi et al., 2017), frond (Hussin et al., 2014; Sa’don et al., 2017), and trunk (Mohtar et al., 2015). These renewable resources are converted into value-added products after a series of treatment processes targeted for lignin removal. Others, such as the leaf and root are poorly characterized despite their importance in feedstock digestibility and BSR pathogenesis studies, respectively. BSR establishes via proximate root-pathogen interaction (Sundram et al., 2014). The white rot fungus, breaches into host cell wall to access nutrients that are available in the starch reservoir (Rees et al., 2009). Lignin, together with other chemical compounds such as suberin and phenolic are first line barriers at the event of pathogen penetration (Boudet, 2000; Donaldson, 2001; Boerjan et al., 2003; Vanholme et al., 2008). For instance, lignin biosynthesis induced by the host cell in response to fungal colonization has been reported in banana roots (Ana and Ian, 2000), wheat cells (Tiburzy and Reisener, 1990) and *Camelina sativa* (Eynck et al., 2012). The extent of lignification in plant tissues is tightly regulated at the transcriptional level (Rogers and Campbell, 2004; Yang et al., 2007). The roles of lignin in plant development have been explicitly characterized; lignin supports the upright positioning of vascular plants and aids water transport system in plants (Sarkanen and Ludwig, 1971; Boerjan et al., 2003; Vanholme et al., 2008). As for lignin in plant defense response, the putative function varies greatly in different plants and thus, remains a subject of major interest, especially in the quest for resistance.

In the present study, oil palm defense responses with respect to lignin and lignin-related biosynthetic pathway were investigated using a popular commercial variety (Dura × Pisifera). Lignification in oil palm seedlings (root tissues) during *G. boninense* infection was quantified at different disease severities under a 12-week disease trial performed in a greenhouse. The control (non-inoculated) and infected (*G. boninense*-inoculated) seedlings were screened for lignin content, lignin composition and the phenylpropanoid pathway enzyme activities (PAL, CAD, and POD). The expression patterns of lignin biosynthetic genes (PAL, CAD, and POD) in root tissues of oil palm seedlings were determined at 1, 2, and 3 wpi using real-time quantitative polymerase chain reaction (RT-qPCR) to understand the influence of phenylpropanoid-regulated genes on host lignification at the transcriptional level.

## Materials and Methods

### Oil Palm Growth

Three month-old oil palm seedlings from the commercial variety Dura × Pisifera (D×P) were obtained from Sime Darby Seeds and Agricultural Services Sdn. Bhd., Selangor, Malaysia. Each seedling was transplanted into a 5 L plastic pot, containing about 3.5 kg of sterilized potting medium (3:2:1 v/v/v topsoil:peat:sand). All seedlings were maintained in a greenhouse (relative humidity of 90 ± 5% and temperature of 33 ± 5°C) for 30 days (approximately 2–3 fully expanded leaves stage) and watered daily.

### Inoculation Procedure

A pathogenic *G. boninense* strain (T10) obtained from Applied Agricultural Resources, Sdn. Bhd., Sungai Buloh, Malaysia was used to inoculate the seedlings. A fungal culture grown on potato dextrose agar (PDA) (Difco, Germany) was subjected to PCR verification using our in-house primers which amplify ITS regions specific to *G. boninense* (Govender et al., 2016). For large-scale inoculum preparation, the primary culture was inoculated on maltose extract agar (MEA) (Merck, Germany) plates to obtain secondary cultures. Rubber (*Hevea brasiliensis*) wood block (RWB) measuring 6 cm × 6 cm × 6 cm was air dried under the hot sun, soaked in distilled water for 24 h, and then sterilized at 121°C for 15 min. This step was repeated. Thereafter, approximately 100 ml of MEA was swirled evenly over the surfaces of each RWB. The MEA treated-RWBs were sterilized again and cooled to room temperature (28 ± 2°C). Following sub-culturing, the 7-day-old fungal culture was used for subsequent RWB inoculations. Each RWB was inoculated with three pieces of mycelial cubes (1 cm × 1 cm × 1 cm), with each individual cube macerated at three different sides of the RWB. The inoculated RWBs were incubated in a dark chamber for 4 weeks at room temperature (Govender et al., 2016). Only RWBs showing active colonization (all RWB surfaces completely covered by mycelia) were used for artificial infection of oil palm seedlings. For the disease trial, each seedling was carefully excavated from the potting media and the bole was then placed directly on top of a fully colonized RWB inoculum. Next, the roots were dispersed to the sides of the RWB (Nur Ain Izzati and Abdullah, 2008). The seedling together with the attached RWB inoculum, was replanted into the potting media.

### Experimental Design

An experiment of two treatments (control and infected) with four biological replicates per treatment and seven sampling times at 1, 2, 3, 4, 8, and 12 weeks post-inoculation (wpi) was arranged in a completely randomized design (CRD). Root tissues of the control (non-inoculated) and infected (inoculated) seedlings collected at 1, 2, and 3 wpi were used for gene expression study, while those collected at 4, 8, and 12 wpi were used for lignin content, lignin composition, enzyme activities (PAL, CAD, and POD), growth (height and weight) and disease assessment.

#### Plant Growth and Disease Assessment

Height and fresh weight of the oil palm seedlings were measured using a tape and digital weighing scale, respectively. Disease severity (DS) was recorded in accordance with a sequential disease development scale (Nur Ain Izzati and Abdullah, 2008). Disease symptoms were scored as follows; 0 = healthy seedling (in absence of yellowing leaf and hyphal mass formation on root surface), 1 = in absence of yellowing leaf and visible formation of hyphal mass on the root surface, 2 = at least 2 or more yellowing leaves with hyphal mass formation on the root surface, 3 = 3 or more yellowing leaves and at least 50% root necrosis with/without sporophore at the bole, 4 = browning of all leaves, 80% root necrosis with/without basidiocarp at the bole. DS (%) was expressed as average score obtained from four biological replicates.

#### Biochemical Analysis

##### Extractive-free root tissue preparation

Root tissues dried at 80°C for 48 h were pulverized into fine powder using a blender (Kenwood BL227). About 200 mg of the powdered root was extracted with 20 ml of washing buffer (100 mM K_2_HPO_4_/KH_2_PO_4_, pH 7.4, 0.5% Triton X-100), followed by 95% methanol. The extraction was repeated until colorless extractive solvent with zero absorbance at 280 nm was obtained. The extractive-free sample was dried at room temperature prior to lignin content and composition analyses.

##### Determination of lignin content

The content of thioglycolic acid (TGA) lignin in oil palm root tissues was determined according to a method by Bruce and West (1989), with several modifications. The extractive-free root powder (2 mg) treated with 1.5 ml of 2 N HCl and 0.3 ml TGA was incubated at 95°C for 4 h with regular shaking at 150 rpm. The mixture was rapidly cooled on ice, and then centrifuged at 15,000 *g* for 10 min. The supernatant was discarded. The pellet was washed thrice with 1 ml of distilled water prior to addition of 1 ml of 0.5 N NaOH. Samples were incubated at room temperature for 18 h with regular shaking (150 rpm). Centrifugation was repeated. The resultant supernatant treated with 0.3 ml of concentrated HCl was incubated at 4°C for an overnight to precipitate the lignothioglycolate derivatives. Centrifugation was repeated and the supernatant was discarded. The resultant pellet was solubilised in 1 ml of 0.5 N NaOH and absorbance was measured using a MultiScan Go spectrophotometer (Thermo Fisher Scientific, United States) at 280 nm. Lignin content expressed in concentration was calculated using an extinction coefficient of 0.513 × 10^-12^ (cm^-1^) (M^-1^) derived from commercial alkali lignin (Sigma–Aldrich, United States).

##### Determination of lignin composition

Lignin composition was determined using the nitrobenzene oxidation method described by Lin and Dence, 1992 with slight modifications. The dried lignin residue (obtained from the nitrobenzene oxidation procedure) suspended into acetonitrile:water (1:1) solution was analyzed by High-Performance Liquid Chromatography (HPLC) using the Luna C5 (5 μm × 150 × 4.6 mm) column/Part No: OOF-4043-EO coupled with a C5 Security Guard Cartridge (Phenomenex, United States). The injector and column temperature was set at 25°C and the injection volume was set at 10 μl. The mobile phase was 0.5% of trifluoroacetic acid in water/acetonitrile (6:1). Run time was set at 30 min while the flow rate was 1 ml per min. All compounds were identified at 280 nm and quantified by comparison with the retention times and the UV absorbance spectra of vanillin and syringaldehyde standards (Sigma–Aldrich, United States).

#### Determination of Enzyme Activities

Fresh root samples were ground into fine powder with liquid nitrogen using a sterilized pestle and mortar. Fine powders (0.2 g) were homogenized in an ice-cold extraction buffer (15 ml) as described by Ana and Ian (2000). The concentration of PAL, CAD, and POD in the supernatant (0.2 ml) was measured at 290, 340, and 470 nm, respectively, using a MultiScan Go (Thermo Fisher Scientific, United States) spectrophotometer. Concentrations were calculated for every 0.01 g of root tissues based on an extinction coefficient (€) value derived as follows: PAL (€) = 2.6323 × 10^-6^ (M^-1^) (cm^-1^); CAD (€) = 2.5685 × 10^-6^ (M^-1^) (cm^-1^); and POD (€) = 1.2349 × 10^-6^ (M^-1^) (cm^-1^). The (€) value of PAL, CAD, and POD was generated using L-phenylalanine, sinapyl alcohol and guaiacol, respectively (Sigma–Aldrich, United States).

#### Relative Quantification by qPCR

Root samples collected at 1, 2, and 3 wpi were flash frozen in liquid nitrogen and stored at -80°C until further use. Total RNA was isolated using a GeneAid RNA Extraction Kit (Thermo Fisher Scientific, United States) following the manufacturer’s instruction. The concentration and quality of the RNA were determined using a NanoDrop 1000 Spectrophotometer (Thermo Fisher Scientific, United States). The first strand cDNA synthesized using a Maxima Kit (Thermo Fisher Scientific, United States) as described by the manufacturer was used as the template for qPCR. All reactions were performed using a CFX96 Touch^TM^ Real-Time PCR Detection System (BIO-RAD, United States) along with RealMOD^TM^ SYBR Green PCR Master Mix (NHK, Bioneer, South Korea) following the manufacturer’s protocol. The β-actin gene was used to normalize the expression of target genes in the infected seedlings relative to the controls. All primers were designed using the Primer 3 software (**Table 1**). The conditions for qPCR were set as followings: initial denaturation at 94°C for 5 min, 94°C for 5 s and 60°C for 30 s (35 cycles) and dissociation at 94°C for 15 s. Each sample contained 10 ng of cDNA and 0.2 μM of each primer in a 20 μl final reaction. Each target gene was calibrated against actin reference gene and quantification of transcripts was presented as 2^-(Δ ΔC_T_)^ in accordance with the *C*_T_ method (Livak and Schmittgen, 2001). All genes were analyzed in three technical replicates and four biological replicates under a similar detection system.

**Table 1:** Description of primers used in quantitative polymerase chain reaction (qPCR) analysis.

Accession number	Gene identity	5′–3′ sequence
EL690110.1	β-Actin (Forward)	5′-GCCTACAATATGGCTGAC-3′
	β-Actin (Reverse)	5′-CCAAGTCATGTAGGGTTG-3′
DW248399.1	POD (Forward)	5′-CTCCTCAATGTCCTCAAC-3′
	POD (Reverse)	5′-GGCAGGTAGGT€GTTC-3′
KM230816	PAL (Forward)	5′-TGTTCGAGGC€CATC-3′
	PAL (Reverse)	5′-GTGGTGCTTCAGTTTGT-3′
EL595475	CAD (Forward)	5′-TCATCCGCACACATTTC-3′
	CAD (Reverse)	5′-GCCATGGCACATATTCT-3′

### Statistical Analysis

One-way analysis of variance (ANOVA) was performed to assess differences in growth (height and fresh weight), lignin content and enzyme (PAL, CAD, and POD) activities in the infected and control seedlings with the PROC GLM procedure using the SAS (version 9.3; SAS Institute) software. In presence of significant difference, the mean separation was performed using Fisher’s protected least significant difference (LSD) method at *p* < 0.05.

## Results

### Disease Profile of Oil Palm Seedlings during *G. boninense* Infection

**FIGURE 1:**
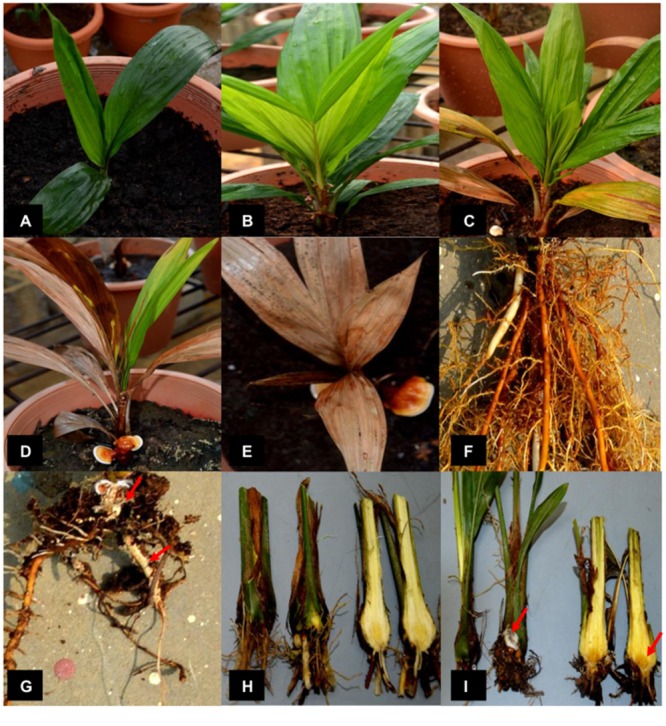
Progression of basal stem rot in 3-month-old oil palm seedlings challenged with *Ganoderma boninense*. **(A)** Control (non-inoculated) seedling. **(B)** Asymptomatic infected (inoculated) seedling at 4 weeks post-inoculation (wpi). **(C)** Infected seedling at 8 wpi with at least 2 or more yellowing leaves. **(D)** Infected seedling at 12 wpi with al least three or more browning leaves and visible fruiting bodies. **(E)** Infected seedling at >12 wpi shows complete necrosis. **(F)** Healthy roots of a control seedling. **(G)** Roots of an infected seedling show colonization by *G. boninense* (red arrows indicate mycelium attachment). **(H)** Boles of the control seedlings and **(I)** boles of the infected seedlings show brown coloration. Red arrow-indicates sporophore (left) and necrosis (right).

At the end of the 12-week disease trial, all infected seedlings showed BSR symptoms while the control seedlings were healthy. Only healthy seedlings with at least three green leaves (**Figure 1A**) were used as biological replicates in this experiment. The DS of the infected seedlings increased gradually over the sampling time points (**Figure 1**). At 4 wpi (DS at 5%), no external symptoms were observed in the infected seedlings (**Figure 1B**), however, root surface examination revealed a low level of fungal attachments (data not shown). At 8 wpi, the DS increased to 35%. The infected seedlings showed yellowing on the lower leaves (**Figure 1C**). At 12 wpi, the DS increased further to 67.5%. The infected seedlings showed yellowing and/or browning on leaves with button-like basidiocarp at the bole (**Figure 1D**). At >12 wpi, the seedlings showed complete leaf browning (**Figure 1E**). At the end of the disease trial, the bole cross section and root tissues of the control and infected seedlings were compared. Root tissues of the control seedlings were visually large and healthy-looking in absence of any lesion (**Figure 1F**), whereas the root tissues of the infected seedlings were relatively small and mycelium attachments were found at random positions (**Figure 1G**). Cross sections of the control bole showed thick succulent tissues with whitish to yellowish colouration (**Figure 1H**) whereas the infected bole showed extensive brown colouration (**Figure 1I**).

### Growth Profile of Oil Palm Seedlings during *G. boninense* Infection

**FIGURE 2:**
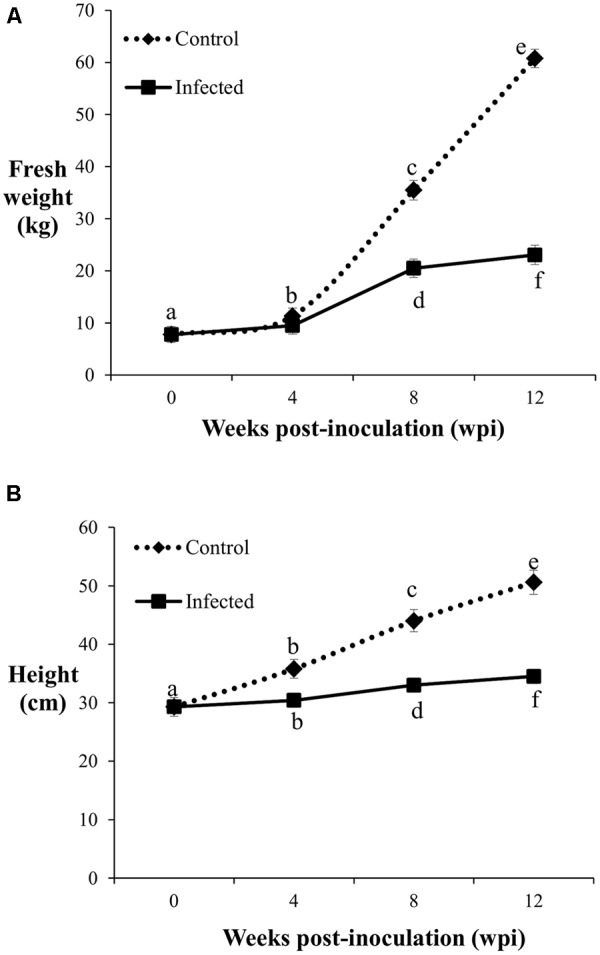
Fresh weight **(A)** and height **(B)** of the control and infected oil palm seedlings challenged with *G. boninense* at 4, 8, and 12 wpi. Bars represent mean ± standard deviation of four biological replicates. Different letters indicate significant difference at *p* < 0.05.

The fresh weight and height of the control and infected oil palm seedlings displayed a similar trend over a period of 12 weeks. The average fresh weight of the control seedlings increased significantly with sampling points. Initially, the average fresh weight of the 4-month-old control seedlings was 7.76 kg and increased by 1.5-fold (11.31 kg) at 4 wpi. At 8 wpi, the average fresh weight of the control seedlings showed a further 3.1-fold increase (35.48 kg), followed by another 2-fold increase (60.76 kg) at 12 wpi (**Figure 2A**). In contrast, the growth rate of the infected seedlings was generally lower than the control seedlings. At 4 wpi, the average fresh weight of the infected seedlings (9.46 kg) showed no significant difference relative to the control seedlings (11.31 kg). The average fresh weight of the infected seedlings increased by 1.9-fold (20.5 kg) at 8 wpi, followed by another 1.1-fold increase (23.04 kg) at 12 wpi.

The average height of the control seedlings was 29.3 cm, increased significantly to 35.8 cm at 4 wpi, followed by further significant increments with 44 cm at 8 wpi and 50.6 cm at 12 wpi (**Figure 2B**). At 4 wpi, the average height of the infected seedlings (30.4 cm) showed no significant difference compared to the control seedlings, while at 8 and 12 wpi, the respective height increased significantly to 33 and 34.5 cm. Significant differences in average height as affected by disease development were notable at 8 wpi onwards. The average height increment at 8 and 12 wpi in the infected seedlings were significantly lower than the corresponding control seedlings (**Figure 2B**). The growth differences (fresh weight and height) observed between the infected and control seedlings at the different sampling points demonstrated BSR disease manifestation.

### Lignin Content and Composition in Oil Palm Roots during *G. boninense* Infection

**FIGURE 3:**
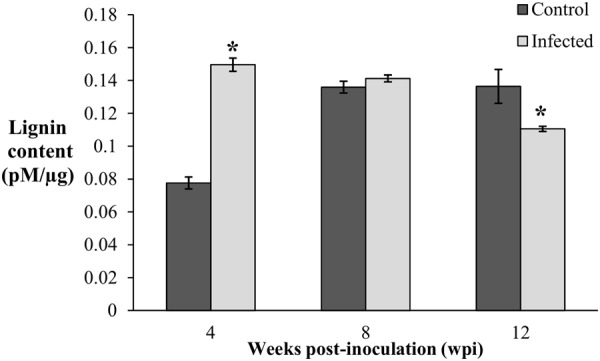
Lignin content in root tissues of the control (non-inoculated) and infected, *Ganoderma boninense* challenged oil palm seedlings at 4, 8, and 12 wpi. Disease severity (DS) of the infected seedlings at 4, 8, and 12 wpi was 5, 34, and 69%, respectively. Bars represent the mean ± standard deviation of four biological replicates. Asterisk indicates significant difference at *p* < 0.05.

Since lignification is regulated during growth, the biosynthesis of induced lignin during disease development was taken into consideration only when the lignin content of the infected seedlings was significantly higher than the control seedlings. Lignin content was significantly high (0.15 pM/μg) in the infected seedlings collected at 4 wpi (DS at 5%) and remained elicited (0.14 pM/μg) with no difference at 8 wpi (DS at 34%). Correspondingly, lignin contents of the control seedlings at 4 and 8 wpi were 0.08 and 0.14 pM/μg, respectively. At the advanced stage of BSR progression, 12 wpi (DS at 69%), lignin content of the infected seedlings decreased to 0.11 pM/μg, while the control seedlings had 0.14 pM/μg (**Figure 3**).

The lignin content of the infected seedlings at 4 wpi (DS at 5%) was significantly higher (0.15 pM/μg) than the control seedlings (0.08 pM/μg) and thus, confirmed the accumulation of disease-associated lignin or induced lignin. The accumulation of lignin at 4 wpi (DS at 5%) also demonstrated the early defense response of oil palm seedlings against *G. boninense*. At 8 wpi (DS at 34%), lignin content of the infected seedlings (0.14 pM/μg) showed no significant difference as compared to the control seedlings (0.14 pM/μg). Reduced lignin content in the infected seedlings at 12 wpi (DS at 69%) implied the occurrence of active colonization by *G. boninense*, which had successfully degraded lignin for nutrient acquisition. The lignin content in the control seedlings was significantly low at 4 wpi and then increased at 8 and 12 wpi. The rise in developmentally regulated lignin in the control seedlings at 4 and 8 wpi, were in parallel to the growth profile; the height and fresh weight at 4 wpi showed significantly lower performance than 8 and 12 wpi (**Figure 2**). Lignin content increase over the sampling time points in the control seedlings corroborated with the growth data (increased height and fresh weight).

To evaluate the monomeric composition of lignin, root tissues from each treatment (control and infected seedlings at different sampling points) were subjected to alkaline nitrobenzene oxidation analysis. Vanillin and syringaldehyde are non-condensed fractions of lignin obtained from this procedure (Chiang and Funaoka, 1988). Both vanillin and syringaldehyde are derived from the guaiacyl and syringyl monomers present in lignin, respectively. The relative abundance of syringyl and guaiacyl monomers is denoted as the S/G ratio. The nitrobenzene oxidation products from root tissues of oil palm seedlings yielded guiacyl and syringyl monomers. The S/G ratio of the control and infected seedlings ranged from 0.42–1.97 to 0.35–0.42, respectively (**Table 2**).

**Table 2:** Lignin composition measured as syringyl to guaiacyl (S/G) ratio in oil palm root tissues; control seedlings (non-inoculated) and infected seedlings (*Ganoderma boninense*-inoculated).

Weeks post-inoculation	Lignin composition^a^ (S/G ratio)	
	Control seedlings	Infected seedlings
0	0.38 ± 0.08	0.37 ± 0.07
4	0.42 ± 0.08	1.31 ± 0.03^b^ (DS at 5%)
8	0.36 ± 0.06	0.64 ± 0.04^b^ (DS at 34%)
12	0.42 ± 0.04	0.5 ± 0.04^b^ (DS at 69%)

*^a^Syringaldehyde (S) and vanillin (G) were obtained from root tissues treated by nitrobenzene oxidation procedure. ^b^Disease severity (DS). Values for lignin composition represent the mean ± standard deviation of results from the analysis of four biological replicates.*

The S/G ratio of the infected seedlings at 4 wpi (DS at 5%) was 1.31, about three times higher than the control seedlings (0.42). The results demonstrated an apparent accumulation of syringyl-enriched lignin in the infected seedlings. As the disease advanced at 8 wpi (DS at 34%), the S/G ratio of the infected seedlings was 0.64, also a two-times increase than the control seedlings (0.36). At 12 wpi (DS at 69%), the S/G ratio of the infected and control seedlings were 0.5 and 0.36, respectively.

### Activities of Phenylpropanoid Pathway Enzymes during *G. boninense* Infection

**FIGURE 4:**
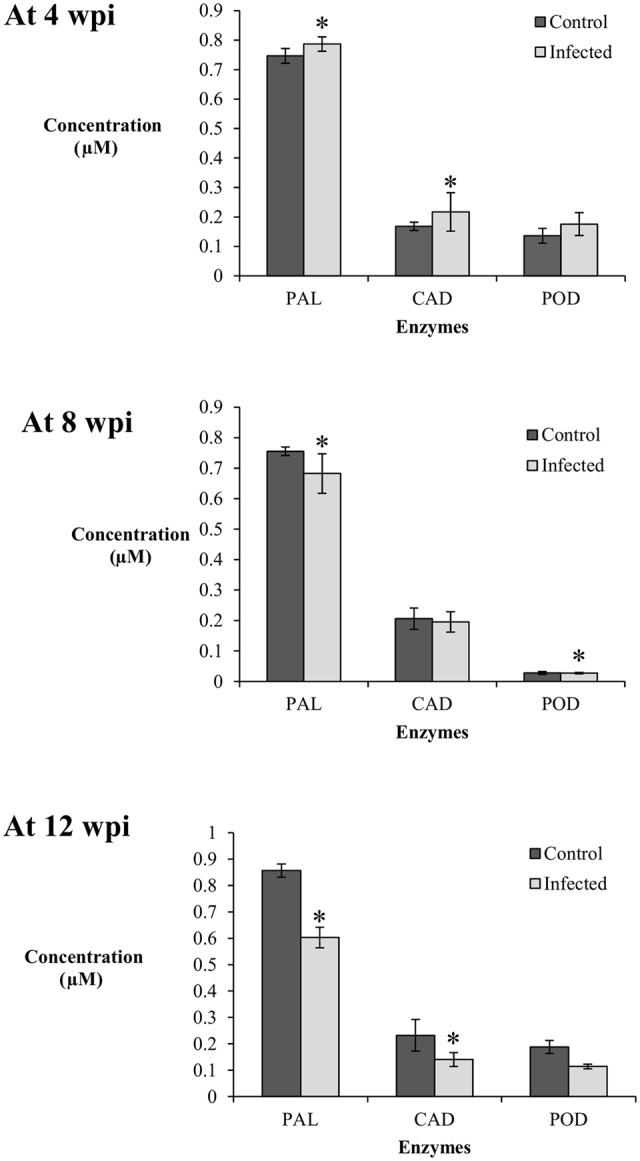
Enzyme activity in the root tissues (0.01 g) of control (non-inoculated) and infected (*Ganoderma boninense*-challenged) oil palm seedlings at 4, 8, and 12 wpi; phenylalanine ammonia lyase (PAL), cinnamyl alcohol dehydrogenase (CAD), and peroxidase (POD). Bars represent mean ± standard deviation of four biological replicates. Asterisk indicates significant difference at *p* < 0.05.

Generally, the PAL enzyme showed the highest activity among the enzymes studied at 4, 8, and 12 wpi; concentration of PAL was four times higher than CAD and POD (**Figure 4**). In the phenylpropanoid pathway, PAL catalyzes the first step of phenylpropanoids biosynthesis, which are then further modified into an array of phenolic compounds. The activity of PAL confirms its role as the precursor for a broad range of phenolics such as flavonoids, tannin, and pectin.

At 4 wpi, the activities of PAL and CAD in the infected seedlings (DS at 5%) were significantly high in comparison to their respective control seedlings, while POD showed no difference between the treatments. The concentrations of PAL and CAD in the control seedlings were 0.75 and 0.17 μM, respectively, whereas their respective concentrations in the infected seedlings were 0.79 and 0.22 μM. At 8 wpi (DS at 34%), the activity of PAL was significantly higher in the control seedlings (0.76 μM) in comparison to the infected seedlings (0.68 μM). The activity of CAD showed no difference between the treatments while the activity of POD in the infected seedlings decreased significantly (0.14 μM) as compared to the control seedlings (0.16 μM). At 12 wpi, both PAL and CAD displayed a similar trend; the enzyme activities in the infected seedlings (DS at 69%) were significantly lower than the control seedlings. The PAL and CAD concentrations in the infected seedlings decreased to 0.60 and 0.14 μM, respectively, whereas the concentrations of the control seedlings were 0.86 and 0.23 μM, respectively. POD activity showed no significant difference between the infected and control seedlings (**Figure 4**).

Elicitation of PAL and CAD activities in the infected seedlings at 4 wpi (DS at 5%) were parallel to the accumulation of induced lignin, suggesting both enzymes may have exerted a collective role in the biosynthesis of induced lignin. CAD, responsible for monolignol production, could be closely linked to the concomitant biosynthesis of S-enriched lignin. The POD activity in the infected seedlings showed no apparent elicitation as compared to the control seedlings at 4, 8, and 12 wpi. Therefore, polymerization of monolignols by POD could not be related to the biosynthesis of induced lignin in this study.

### Expression of Phenylpropanoid Pathway Genes during *Ganoderma boninense* Infection

**FIGURE 5:**
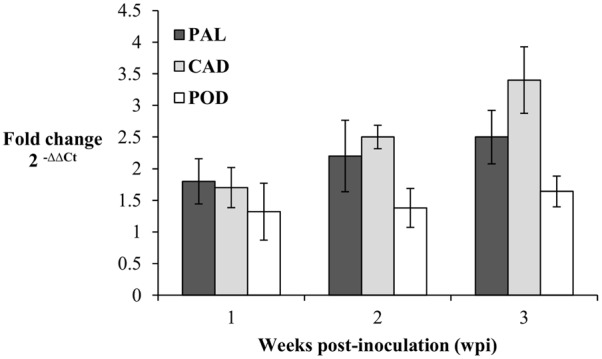
Expression pattern of PAL, CAD, and POD genes in *Ganoderma boninense* infected oil palm seedlings at 1, 2, and 3 wpi. Fold change (FC) values are computed relative to control (non-inoculated) seedlings. Bars represent mean ± standard error of four biological replicates.

The expression patterns of PAL, CAD, and POD genes in the root tissues of oil palm seedlings were profiled at 1, 2, and 3 wpi. Transcript levels in the infected seedlings were measured as fold-change (FC) relative to the control seedlings according to Livak and Schmittgen (2001). At 1 wpi, the transcript levels of PAL, CAD, and POD in the infected seedlings were up-regulated, at 1.8, 1.7, and 1.3 FC, respectively. Similarly, at 2 wpi, the transcript levels of PAL, CAD, and POD increased further to 2, 2.1, and 1.4 FC, respectively, and at 3 wpi, the transcript levels PAL, CAD, and POD genes were 2.5, 3.2, and 1.6 FC, respectively. In brief, throughout the sampling points (1–3 wpi), each gene showed gradual increases in transcript levels. The CAD gene had the highest transcript abundance, followed by PAL and POD genes at 3 wpi (**Figure 5**).

## Discussion

### Lignin Content and Composition in Oil Palm Roots during *Ganoderma boninense* Infection

The cell walls of plants contain cellulose, hemicellulose, and lignin. The content and composition of these polymers vary among the different plant organs and within a specific tissue in the plants (Zaprometov et al., 1993; Campbell and Sederoff, 1996; Schuetz et al., 2013; Lourenço et al., 2016). In addition, lignin variation is affected by environmental conditions and plant growth stages (Ralph and Hatfield, 1991; Buranov and Mazza, 2008). In our study, the BSR development was characterized into three distinct stages. In the first stage, the asymptomatic plants showed low level of fungal attachment on the root surfaces, later at the second stage, the yellowing leaves indicated exhaustion of starch reservoirs resulting from biotrophic interaction of the pathogen at the expense of host and in the final stage (necrosis), the whole plant became affected, with leaves and stems turning brittle prior to plant death. The observation was comparable with previously reported findings (Flood et al., 2003; Rees et al., 2009). Oil palm early defense response at 4 wpi (DS at 5%) showed accumulation of lignin at two-fold higher than the control seedlings. In addition, the accumulated induced lignin showed a higher syringyl (S) to guaiacyl (G) ratio (S/G) in the infected seedlings compared to the control seedlings, demonstrating that S-lignin was selectively synthesized by the host plant during the *G. boninense* infection. Although such findings have been commonly observed in many plant–pathogen interactions (Menden et al., 2007; Moura et al., 2010; Barros-Rios et al., 2011; Bi et al., 2011; Eynck et al., 2012; Cheng et al., 2013; Ali and Mc Near, 2014; Bagniewska-Zadworna et al., 2014; Bivi et al., 2014), our study is the first to report concomitant alteration of lignin content and composition (S/G ratio) in *G. boninense* infected oil palm seedlings. Lignin accumulation at 4 wpi (DS at 5%) may have limited the host cell wall digestibility and restricted *G. boninense* infection. Despite a significant accumulation of lignin at 4 wpi (DS at 5%), the host plant was succumbed to attack at 8 wpi (DS at 34%) and 12 wpi (DS at 69%) and subsequently died.

At 4 wpi (DS at 5%) and 8 wpi (DS at 34%), the biosynthesis of S monomers was higher than G monomers, as indicated by the increased S/G ratio in the infected seedlings. The accumulation of either S or G monomers has been reported to be species-specific (Novo-Uzal et al., 2012). In carnation, the G-S lignin in control plants changed to mainly G-rich lignin during defense response against *Fusarium oxysporum* (Steijl et al., 1999). Contrarily, induced lignin with significant accumulation of S monomers has been reported during disease development in wheat (Tiburzy and Reisener, 1990; Mitchell et al., 1999).

Lignin polymers are chemically flexible, that the monomeric variation is affected by cell and environmental conditions (Faix et al., 1985; Boudet, 2000). A decrease in G monomers may imply a preferential degradation of G monomers compared to S monomers in oil palm root tissues colonized by *G. boninense*. Several studies have closely linked degradation of lignin to the types of monomer present in the wood tissues (Highley, 1982; Blanchette, 1991; Syafii and Yoshimoto, 1991; Skyba et al., 2013). Both the S and G monomers differ in the rate of methoxylation. The former is methoxylated at the 3′ and 5′ position of the aromatic ring while the latter carries only one methoxyl at the 3′ position, leaving the 5′ position free for participation in branching reactions (Sarkanen and Ludwig, 1971). A linear structured lignin is more recalcitrant to oxidizing agents compared to a branched structural pattern (Stewart et al., 2009). Biosynthesis of lignin is controlled by a complex phenylpropanoid biochemical grid, nevertheless, the regulatory roles posed by each member in the pathway cumulatively fix the composition and content of lignin synthesized at any given time (Rogers and Campbell, 2004; Yang et al., 2007). An increase in the S-rich lignin also implies the need to understand other specific enzymes such as ferulic acid 5-hydroxylase (F5H) and 5-hydroxyferulic acid o-methyltransferase (COMT), which catalyze the formation of sinapaldehyde, which in turn results in the biosynthesis of S monolignols. Further studies on enzymes specific to S monomer lignin need to be taken up to comprehend the inter-play between the members of the complex phenylpropanoid pathway.

### Transcript Levels of Phenylpropanoid Pathway Genes during *Ganoderma boninense* Infection

In the present study, an increase in total lignin content with enriched S monomer at 4 wpi (DS at 5%) was preceded by up-regulation of PAL, CAD, and POD genes at 1, 2, and 3 wpi. In addition, the activities of enzymes in the phenylpropanoid pathway were in parallel to the gene expression patterns. Knock-out studies conducted in *Pinus taeda* (Mackay et al., 1997) and *Medicago sativa* (Baucher et al., 1999) have demonstrated the role of CAD in modulating lignin composition and content. A reduced expression of CAD in a mutant *P. taeda* significantly decreased the total lignin content while an increased expression correspondingly increased the total lignin content. Likewise, a reduction in the CAD activity observed in *M. sativa* yielded a lower syringyl to guaiacyl (S/G) ratio in comparison to the control (Baucher et al., 1999). Extensive studies performed on transgenic species with lost CAD activity had demonstrated intense changes in lignin composition rather than lignin content (Yahiahui et al., 1998; Eudes et al., 2006). The present results demonstrated the role of CAD in regulating defense associated-lignin, composed of predominantly, the S monomer. The results were comparable to other similar studies in forage grass (Giordano et al., 2014), tobacco (Chabannes et al., 2001), wheat (Mitchell et al., 1999), and Arabidopsis (Tronchet et al., 2010).

Phenylalanine is the precursor in the complex phenylpropanoid pathway (Mitchell and Walters, 1995; Kato et al., 2000; Zang et al., 2015). In the present study, PAL, together with CAD and POD genes, were collectively up-regulated at the early stage of *Ganoderma* infection (1, 2, and 3 wpi). Among the three genes, the transcript level of PAL was highest at 1 wpi, however, at 2 and 3 wpi, CAD consistently showed the highest expression. The transcript level of POD gene was the lowest at 1, 2, and 3 wpi. The specificity of lignin polymerization has been reported to exhibit a spatiotemporal expression pattern (Francoz et al., 2014). Peroxidase and laccase genes polymerise individual monolignols to form the complex lignin (Fagerstedt et al., 2010; Berthet et al., 2012). While laccase had been reported to display G-specific polymerization (Berthet et al., 2011), contrarily, the peroxidase showed a broader selectivity; polymerization of p-hydroxyphenyl (H), syringyl (S), and guaiacyl (G). The peroxidase comprises a group of multiple isoenzymes with adverse substrate specification for polymerization. In this study, the isoform of the peroxidase gene studied may not play a critical role in the biosynthesis of S-enriched lignin in oil palm seedlings during defense response against *Ganoderma*. The monolignol production evaluated by transcript abundance of CAD was ultimately higher than the degree of polymerization (transcript abundance of POD). The results were in agreement with Barrière et al., 2015, whom demonstrated that the multigene families responsible for monolignol polymerizations in maize could not be considered as a significant determinant factor affecting lignin variation. Further studies evaluating each member of the oil palm peroxidase multigene family need to be carried out to better comprehend the role of peroxidases in the production of S-lignin during *G. boninense* root colonization.

### Potential Modifications of Lignin in Oil Palm Roots

In the present research, we demonstrated rapid lignification with increased S/G ratio in oil palm seedlings (root tissues) as a possible defense response against *Ganoderma* infection. We also identified PAL and CAD as potential phenylpropanoid-biosynthetic enzymes and genes involved in regulating the biosynthesis of S-enriched lignin during the early stage of *Ganoderma* infection. In the quest for efficient BSR management strategy, further studies on host lignin compositional difference and its influence on *Ganoderma* infection need to be undertaken at a large scale. Phenomic studies which integrate agronomic and lignin data should be conducted to understand the physicochemical relationships of these parameters. Based on these results, PAL and CAD, in particular, may potentially be targets for up-regulation, ideally to confer S-enriched defense lignin.

## Author Contributions

NTG conducted the experiment, analyses and wrote the manuscript. IAS, MM, and M-YW co-ordinated the work and secured fundings.

## Conflict of Interest Statement

The authors declare thatthe research was conducted in the absence of any commercial or financial relationships that could be construed as a potential conflict of interest.
